# Standardized Beating-Heart Aortic Arch Reconstruction with Simultaneous Cerebral and Coronary Perfusion in Neonates and Infants: A Single-Center Cardiovascular Cohort Study

**DOI:** 10.3390/jcdd13040161

**Published:** 2026-04-07

**Authors:** Shiraslan Bakhshaliyev, Ergin Arslanoglu

**Affiliations:** 1 Pediatric Cardiovascular Surgery Department, Atlas University Hospital, Istanbul 34203, Turkey; erginarslanoglu@gmail.com; 2 Pediatric Cardiovascular Surgery Department, Liv Bona Dea Hospital, Baku 1060, Azerbaijan

**Keywords:** aortic arch reconstruction, congenital heart disease, neonatal cardiac surgery, antegrade selective cerebral perfusion, coronary perfusion, aortic coarctation

## Abstract

Background: Neonatal and infant aortic arch reconstruction remains a high-risk cardiovascular procedure requiring effective cerebral and myocardial protection. Variability in perfusion strategies may influence early hemodynamic stability and postoperative recovery. This study aimed to evaluate the early and short-term cardiovascular outcomes of a standardized beating-heart aortic arch reconstruction strategy incorporating simultaneous antegrade selective cerebral and continuous coronary perfusion. Methods: In this retrospective single-center cohort study, 31 consecutive neonates and infants undergoing aortic arch reconstruction between November 2022 and December 2025 were analyzed. A standardized surgical protocol was applied, consisting of extensive ductal tissue resection, interdigitating posterior end-to-end anastomosis, anterior autologous pericardial patch augmentation, and moderate hypothermic antegrade selective cerebral perfusion combined with continuous coronary perfusion via innominate artery cannulation. Early postoperative outcomes and short-term echocardiographic follow-up results were assessed. Results: The cohort included 31 patients, 22.6% of whom had complex associated cardiac anomalies requiring concomitant procedures. Median cardiopulmonary bypass and aortic cross-clamp times were 119 and 64 min, respectively. There was no in-hospital mortality. Major complications were infrequent, and median intensive care unit stay was 5 days. During a median follow-up of 6.8 months, one patient (3.2%) developed recoarctation requiring reintervention. No late mortality was observed. Conclusions: A fully standardized beating-heart aortic arch reconstruction strategy incorporating simultaneous cerebral and coronary perfusion demonstrated favorable early cardiovascular and short-term outcomes, even in anatomically complex cases. Preservation of continuous coronary perfusion may be associated with improved myocardial stability and early postoperative recovery; however, these findings should be interpreted as observational and hypothesis-generating given the absence of a control group. Larger multicenter studies with longer follow-up are warranted to confirm these findings.

## 1. Introduction

Aortic arch pathologies constitute one of the most challenging areas of congenital cardiac surgery, particularly in the neonatal and infant age groups. Lesions such as aortic arch hypoplasia, coarctation of the aorta, and interrupted aortic arch are frequently encountered in association with additional cardiac anomalies, which further increases the complexity of the surgical strategy. The primary objectives of surgery in this patient population are to ensure safe cerebral protection, to achieve reconstruction of the aortic arch with adequate diameter and geometric integrity, and to minimize the risk of late recoarctation [1,2].

In neonatal and infant aortic arch reconstruction, there is still no consensus in the literature regarding the optimal surgical technique and perfusion strategy. Although deep hypothermic circulatory arrest (DHCA) has been used as the standard approach for many years, its application has become more limited in recent years because of potential risks associated with cerebral and systemic organ injury [3]. In this context, antegrade selective cerebral perfusion (ASSP/ACP) techniques have emerged as alternative strategies aimed at improving neurological outcomes by maintaining the continuity of cerebral blood flow [4].

Clinical series and meta-analyses published in recent years have demonstrated that ACP provides safe cerebral protection, particularly in complex aortic arch reconstructions requiring prolonged perfusion times. Nevertheless, issues such as the optimal perfusion temperature, flow rate, and the necessity of concomitant coronary perfusion remain subjects of ongoing debate [5]. In particular, ACP performed on the beating heart has been suggested to reduce the risk of myocardial ischemia and to support early postoperative hemodynamic stability [6].

From a surgical perspective, resection of the coarcted segment followed by posterior end-to-end anastomosis and anterior arch augmentation represents a widely used approach aimed at preserving the anatomical continuity and growth potential of the aortic arch.

The use of autologous pericardium as an anterior patch material has been reported to be an advantageous option in terms of infection risk, foreign body reaction, and the development of long-term stenosis [7]. Despite this, significant heterogeneity exists among centers with regard to technical details and the materials used.

A substantial proportion of studies in the literature consist of multicenter series that include heterogeneous surgical techniques and perfusion strategies. This heterogeneity makes it difficult to accurately evaluate the true effectiveness of a specific surgical approach and cerebral protection strategy. Therefore, presenting outcomes obtained using a standardized surgical technique and perfusion protocol may provide a meaningful contribution to the existing literature.

In this study, we aimed to present the early and short-term outcomes of a standardized aortic arch reconstruction technique—consisting of resection of the coarcted segment, posterior end-to-end anastomosis, and anterior augmentation with an autologous pericardial patch—performed under beating-heart antegrade selective cerebral perfusion in neonatal and infant patients. The objective of this study was to evaluate the safety, feasibility, and early clinical outcomes of this approach.

## 2. Materials and Methods

This study is a single-center retrospective cohort study evaluating neonatal and infant patients who underwent aortic arch reconstruction at our institution. The study protocol was approved by the Institutional Ethics Committee (Date: 1 July 2025; Protocol No: 184), and the research was conducted in accordance with the principles of the Declaration of Helsinki and Good Clinical Practice guidelines. The study was reported in accordance with the STROBE guidelines for observational studies.

Neonatal (<28 days) and infant (≥28 days) patients who underwent aortic arch reconstruction at our center between November 2022 and December 2025 were included in the study.

The inclusion criteria were defined as having undergone aortic arch reconstruction via median sternotomy for aortic coarctation, aortic arch hypoplasia (defined as a transverse arch z-score less than −2 or an absolute diameter smaller than body weight +1 mm), or associated cardiac anomalies accompanying these pathologies. Patients who underwent surgery via left thoracotomy for isolated coarctation and those who had previously undergone aortic arch repair using a different surgical technique were excluded from the study. The final analysis was performed on 31 patients.

### 2.1. Preoperative Evaluation

All patients underwent contrast-enhanced computed tomography (CT) angiography prior to surgical planning. CT angiography was used to evaluate in detail the length of the coarcted segment, its relationship with the ductus arteriosus, aortic arch morphology, and particularly the presence of proximal transverse arch hypoplasia. Aortic diameter measurements were obtained at the levels of the ascending aorta, proximal transverse arch, distal transverse arch, and isthmus. These measurements were standardized according to the patients’ body surface area, Z-scores were calculated according to established pediatric normative reference data, and they were used to grade the severity of aortic arch hypoplasia. In all patients identified as having proximal transverse arch hypoplasia, aortic arch reconstruction via median sternotomy was preferred as the surgical approach. Based on preoperative echocardiography, CT angiography, and intraoperative findings, patients were classified into three groups:

- Isolated aortic arch pathology,
- Aortic arch pathology with ventricular septal defect,
- Complex anatomy (such as transposition of the great arteries, double outlet right ventricle, or single-ventricle physiology).

### 2.2. Surgical Technique and Perfusion Strategy

In all patients, the surgical procedure was performed via median sternotomy using a standardized surgical technique and perfusion protocol. Arterial cannulation was performed directly from the innominate artery in all patients. Bicaval cannulation was used in patients requiring intracardiac procedures, whereas single atrial cannulation was performed in patients undergoing isolated aortic arch repair. In all cases, a ventricular vent cannula was placed through the right superior pulmonary vein to prevent ventricular distension during beating-heart surgery. For cerebral protection, beating-heart antegrade selective cerebral perfusion was applied during aortic arch reconstruction at a temperature of 28 °C. In the absence of cerebral near-infrared spectroscopy (NIRS) monitoring, right radial arterial pressure was used as a surrogate parameter to assess the adequacy of cerebral perfusion. During antegrade selective cerebral perfusion, flow was maintained at 40–60 mL/kg/min, and perfusion adequacy was evaluated using right radial arterial pressure monitoring in combination with intraoperative venous blood gas assessment, particularly during the initial phase of perfusion. To maintain continuous coronary perfusion, a 3/16-inch side branch originating from the aortic cannula was connected to the aortic root cannula, thereby providing selective coronary perfusion.

The adequacy of coronary perfusion was closely monitored throughout the operation by direct myocardial inspection and continuous electrocardiographic monitoring. In all patients, the coarcted segment was extensively resected together with ductus arteriosus tissue. The posterior wall of the aortic arch was reconstructed using an end-to-end anastomosis with the interdigitating technique. The anterior wall of the arch was augmented and enlarged using an autologous pericardial patch. This approach aimed to restore anatomical continuity of the aortic arch and preserve long-term growth potential. Associated cardiac anomalies (such as ventricular septal defect) were repaired during the same surgical session using standard surgical techniques.

### 2.3. Data Collection and Definitions

Demographic data, preoperative clinical characteristics, echocardiographic measurements, operative times, and early postoperative outcomes were obtained from patient records and the electronic medical record system.

Major morbidity was defined as the presence of any of the following complications: neurological events, acute kidney injury or the need for peritoneal dialysis, cardiopulmonary resuscitation, requirement for mechanical circulatory support, or recurrent laryngeal nerve injury. Early mortality was defined as death occurring during the index hospital stay.

Postoperative follow-up was performed using clinical evaluation and transthoracic echocardiography. Recoarctation was defined as the presence of a systolic pressure gradient ≥ 20 mmHg measured at the level of the aortic arch or isthmus. After discharge, patients were invited for regular outpatient follow-up visits, and long-term outcomes were monitored using clinical and echocardiographic findings. The date of the most recent echocardiographic examination, aortic arch pressure gradients, and any requirement for reintervention or reoperation were evaluated based on patient records and follow-up data.

Neurological events were defined as any clinically evident neurological deficit or abnormal neurological examination during the postoperative period. Neurological assessment was primarily based on clinical evaluation. Cerebral near-infrared spectroscopy (NIRS) monitoring was not routinely available and therefore was not used in this cohort. Neuroimaging was performed only in patients with clinical suspicion of neurological events and was not routinely applied.

Prolonged ventilation was defined as the requirement for mechanical ventilation exceeding 72 h postoperatively.

As a retrospective single-center study, potential selection and information bias were minimized by including all consecutive eligible patients and using standardized data collection protocols.

### 2.4. Statistical Analysis

Statistical analyses were performed using SPSS version 30 (IBM Corp., Armonk, NY, USA). Continuous variables were presented as median and interquartile range (IQR). Categorical variables were expressed as counts and percentages. Comparisons between neonatal and infant groups were performed using the Mann–Whitney U test for continuous variables and Fisher’s exact test for categorical variables. A *p* value < 0.05 was considered statistically significant. All consecutive eligible patients during the study period were included. No formal sample size calculation was performed due to the exploratory nature of the study.

## 3. Results

A total of 31 neonatal and infant patients were included, of whom 22.6% had complex cardiac anatomy, defined as the presence of major associated congenital cardiac anomalies such as transposition of the great arteries (TGA), double outlet right ventricle (DORV), or single-ventricle physiology, requiring advanced concomitant procedures. Of the patients, 51.6% were neonates, and the median age at the time of surgery was 23 days (IQR, 6–60 days). Patient characteristics and preoperative clinical findings are summarized in Table 1. In more than half of the cases, a ventricular septal defect accompanied the aortic arch pathology (51.6%), while 22.6% of patients exhibited complex anatomical features.

In all patients, the surgical procedure was performed via median sternotomy. As the perfusion strategy, beating-heart antegrade selective cerebral perfusion was applied in all cases. In patients requiring additional procedures, the operation was continued under cardiac arrest. Operative and perfusion times are presented in Table 2. The median cardiopulmonary bypass time was 120.0 min, and the median selective cerebral perfusion time was 37.5 min. Cardiac arrest was applied only when necessary, and in these patients, the median arrest time was 46.5 min. Despite prolonged cardiopulmonary bypass times, ventilation duration, intensive care unit stay, and hospital length of stay were observed to be within clinically acceptable ranges when compared with contemporary neonatal aortic arch reconstruction series reported in the literature.

In addition to aortic arch reconstruction, concomitant cardiac surgical procedures were performed in a substantial proportion of patients. The most frequently performed additional procedure was ventricular septal defect closure, which was carried out in 51.6% of patients. Other advanced surgical procedures included pulmonary artery banding (12.9%), aortic valve commissurotomy (12.9%), and interrupted aortic arch repair (12.9%). In three patients (9.7%), an arterial switch operation was performed concomitantly with aortic arch reconstruction, while one patient (3.2%) underwent repair of infracardiac total anomalous pulmonary venous drainage (TAPVD). Less frequently, subaortic stenosis relief (6.5%) and repair of a right pulmonary artery originating from the aorta (3.2%) were performed. The distribution of additional surgical procedures is shown in Table 3.

Early postoperative and follow-up outcomes are summarized in Table 4. No in-hospital mortality was observed. No patient required extracorporeal membrane oxygenation (ECMO); no re-exploration for bleeding was necessary, and no arrhythmia requiring permanent pacemaker implantation was observed. Early major morbidity rates were low; transient neurological complications occurred in one patient; transient complete atrioventricular block occurred in two patients; deep sternal infection developed in one patient, and acute renal failure requiring peritoneal dialysis occurred in two patients. Neuroimaging was performed in one patient due to clinical suspicion, and no radiological abnormalities were detected.

During postoperative follow-up, 90.3% of patients had a final echocardiographic evaluation available. The median follow-up duration was 6.8 months (IQR, 1.9–18.2). During the follow-up period, recoarctation developed in one patient, and balloon angioplasty of the aortic arch was performed as a reintervention due to recoarctation.

When patients who underwent isolated aortic arch surgery were compared with those who had associated cardiac anomalies in addition to aortic arch pathology, cardiopulmonary bypass time, cross-clamp time, ventilation duration, and total hospital length of stay tended to be shorter in the isolated arch group; however, no statistically significant differences were observed; however, these analyses should be considered exploratory given the small sample size (Table 5).

No in-hospital mortality was observed in either group, and recoarctation developed in one patient in the isolated arch surgery group, while no patient required reoperation. Although statistical significance was not reached, numerical trends favored shorter recovery parameters in the isolated arch group. However, numerical trends favored shorter bypass time and recovery parameters in the isolated arch group.

## 4. Discussion

In this study, we evaluated the early and short-term clinical outcomes of a standardized aortic arch reconstruction technique combined with a beating-heart antegrade selective cerebral and coronary perfusion strategy in neonatal and infant patients. Neonatal aortic arch surgery represents one of the most challenging areas of congenital cardiac surgery, owing to the need for effective cerebral and myocardial protection as well as the long-term maintenance of aortic arch patency. The most notable findings of this study include zero in-hospital mortality, a low recoarctation rate, and low major morbidity rates despite high surgical complexity. These results indicate that the applied surgical and perfusion approach is both safe and effective. Outcomes reported in the literature show a wide variability due to significant heterogeneity in surgical techniques and perfusion strategies [8,9].

One of the most critical steps in this type of surgery is preoperative planning. In all patients presenting with suspected aortic coarctation, detailed evaluation of the transverse arch using contrast-enhanced CT angiography is of great importance for accurate determination of the surgical strategy. Both the literature and clinical experience have reported that interventions performed via left thoracotomy in patients with associated aortic arch hypoplasia are linked to residual stenosis and increased early recoarctation rates. Therefore, in our institution, even in cases of mild proximal transverse arch hypoplasia, the preferred approach is aortic arch reconstruction via median sternotomy, and we believe that this strategy has a positive impact on early and short-term arch patency [10]. To our knowledge, this study represents one of the few single-center cohort analyses evaluating a fully standardized beating-heart aortic arch reconstruction strategy incorporating simultaneous cerebral and coronary perfusion across varying levels of anatomical complexity.

The occurrence of recoarctation after aortic arch reconstruction is a major determinant of long-term outcomes. In particular, inadequate resection of the coarcted segment and suboptimal anastomotic geometry have been associated with an increased need for late reintervention [11]. In the present study, extensive resection of the coarcted segment together with ductus arteriosus tissue and the use of an interdigitating end-to-end anastomosis technique on the posterior wall were employed in all patients to achieve reconstruction of the arch with a more physiological geometry. Recent studies have demonstrated that the interdigitating posterior anastomosis technique may positively influence arch patency and reduce reintervention rates [12]. Anterior arch augmentation using an autologous pericardial patch represents a rational approach and is considered to have growth potential based on previous literature; however, this was not directly evaluated in our cohort due to the limited follow-up duration. The favorable effects of autologous pericardium on mid- and long-term outcomes have been supported by numerous single-center series published in recent years [13].

Although deep hypothermic circulatory arrest (DHCA) was considered the standard approach in neonatal aortic arch surgery for many years, its use has become more limited in contemporary practice because of potential adverse effects on neurological and neurodevelopmental outcomes [14]. In this context, antegrade selective cerebral perfusion (ACP) strategies have been increasingly preferred. Clinical studies and meta-analyses comparing DHCA and ACP strategies have reported that ACP provides more effective preservation of cerebral oxygenation and is associated with lower rates of neurological complications [15]. Nevertheless, there is still no clear consensus regarding the optimal application of ACP, including temperature level, perfusion flow, and duration, and significant variability exists among centers [8]. In our practice, ACP is performed at 28 °C with a cerebral perfusion flow of 40–60 mL/kg/min and a right radial arterial pressure target of 70–90 mmHg.

One of the distinguishing features of this study is that cerebral protection was achieved using beating-heart ACP while simultaneously maintaining coronary perfusion. Beating-heart aortic arch reconstruction has previously been reported to reduce the risk of myocardial ischemia and to support early postoperative hemodynamic stability [16]. Similarly, a recently published large series comparing beating-heart ACP and cardiac arrest strategies in isolated aortic arch reconstruction reported comparable early and mid-term outcomes between the two approaches; however, significantly higher rates of early extubation were observed in patients undergoing beating-heart surgery. The acceptable ventilation duration, intensive care unit stay, and hospital length of stay observed in our cohort—which included both isolated and complex aortic arch cases—support the applicability of the beating-heart cerebro-myocardial perfusion approach not only in isolated cases but also in patients with higher surgical complexity [17]. In contrast to strategies relying solely on cerebral perfusion, the addition of continuous coronary perfusion may be associated with improved myocardial stability and facilitate early postoperative recovery; however, given the absence of a control group, these findings should be interpreted cautiously.

In our protocol, direct arterial cannulation of the innominate artery combined with a 3/16-inch side branch from the aortic cannula connected to the aortic root cannula allowed simultaneous and standardized maintenance of cerebral and myocardial perfusion.

The safety of ACP using innominate artery cannulation has also been supported by previously published experimental and clinical studies [18].

In this series, a substantial proportion of patients underwent advanced concomitant cardiac procedures in addition to aortic arch reconstruction, including ventricular septal defect closure, pulmonary artery banding, arterial switch operation, and infracardiac TAPVD repair.

The literature reports that mortality and morbidity rates may be significantly higher in such complex cases. Despite this, when isolated aortic arch cases were compared with those involving associated cardiac anomalies in our study, no significant differences were observed in terms of in-hospital mortality, major complications, or reintervention rates. Although ventilation duration, intensive care unit stay, and hospital length of stay tended to be longer in the complex group, these differences did not reach statistical significance. These findings suggest that the standardized surgical technique and perfusion strategy can be safely applied across different levels of anatomical and surgical complexity [17]. Reported early mortality rates in neonatal aortic arch surgery range between 5% and 15%, and recoarctation rates vary from 5% to 20% depending on technique and patient complexity.

Despite prolonged cardiopulmonary bypass times, the acceptable ventilation duration, intensive care unit stay, and hospital length of stay observed in our study are noteworthy.

This may be considered an indirect indicator of the effectiveness of cerebral and myocardial protection. When compared with postoperative recovery times reported in series using ACP, our results appear to be at least comparable.

## 5. Limitations

The main limitations of this study include its retrospective design, single-center experience, and relatively small sample size. In addition, detailed perioperative biochemical and hemodynamic parameters, including lactate levels, vasoactive inotropic score (VIS), and inotrope duration, were not consistently available due to the retrospective nature of the study. In addition, the follow-up period is limited to the mid-term, and longer follow-up is required to evaluate long-term aortic arch growth and late recoarctation rates. Nevertheless, the high availability of follow-up echocardiographic data and the need for reintervention in only one patient during follow-up represent important findings supporting the effectiveness of the applied approach. In addition, standardized neurological monitoring such as NIRS and routine neuroimaging were not available, and long-term neurodevelopmental outcomes could not be assessed.

## 6. Conclusions

In conclusion, in neonatal and infant aortic arch reconstruction, a standardized surgical technique consisting of extensive ductal resection, interdigitating posterior anastomosis, and anterior augmentation with an autologous pericardial patch—combined with beating-heart ACP and selective coronary perfusion via the innominate artery—appears to be a safe and effective approach in our series, which included complex cases.

Larger series and longer-term follow-up studies are required to more clearly define the impact of this strategy on neurological outcomes and long-term aortic arch patency.

## Figures and Tables

**Table 1:** Patient Characteristics and Preoperative Findings.

Variable	Value
Number of patients, *n*	31
Age (days), median (IQR)	23 (6–60)
Neonates (<28 days), *n* (%)	16 (51.6)
Male sex, *n* (%)	13 (41.9)
Weight (kg), median (IQR)	3.4 (2.8–4.3)
Body surface area (m^2^), median (IQR)	0.22 (0.19–0.26)
Prostaglandin E1 use, *n* (%)	18 (58.1)
Preoperative inotropic support, *n* (%)	7 (22.6)
Single-ventricle physiology, *n* (%)	3 (9.7)
**Aortic arch anatomy**	
– Isolated aortic arch pathology, *n* (%)	8 (25.8)
– Aortic arch pathology with VSD, *n* (%)	16 (51.6)
– Complex anatomy, *n* (%)	7 (22.6)

**Table 2:** Operative and Perfusion Characteristics.

Variable	Value
**Operative and perfusion data**	
Cardiopulmonary bypass time (min), median (IQR)	120.0 (104.5–138.2)
Selective cerebral perfusion time (min), median (IQR)	37.5 (35.8–45.0)
Cross-clamp time (min)	46.5 (29.5–74.0)
Delayed sternal closure, *n* (%)	15/31 (51.7)
Day of sternal closure, median (IQR)	2 (1–3)
**Early postoperative time parameters**	
Ventilation duration (hours), median (IQR)	48.0 (24.0–96.0)
Intensive care unit stay (days), median (IQR)	7.0 (5.0–9.0)
Hospital length of stay (days), median (IQR)	14.0 (10.2–17.5)

**Table 3:** Concomitant Surgical Procedures Performed with Aortic Arch Reconstruction.

Concomitant Surgical Procedure	*n* (%)
Ventricular septal defect closure	16 (51.6)
Pulmonary artery banding	4 (12.9)
Arterial switch operation	3 (9.7)
Aortic valve commissurotomy	4 (12.9)
Interrupted aortic arch repair	4 (12.9)
Infracardiac total anomalous pulmonary venous drainage repair	1 (3.2)
Repair of anomalous origin of the right pulmonary artery from the aorta	1 (3.2)
Relief of subaortic stenosis	2 (6.5)

**Table 4:** Early Postoperative and Follow-Up Outcomes.

Outcome	*n*/Available (%)
**Early postoperative outcomes**	
In-hospital mortality	0/31 (0)
Cardiopulmonary resuscitation	0
Requirement for mechanical circulatory support	0
Neurological event	1 (3.2)
Requirement for peritoneal dialysis	2 (6.4)
Transient complete atrioventricular block	2 (6.4)
Deep sternal infection	1 (3.2)
Prolonged ventilation (>72 h)	3 (9.6)
**Follow-up outcomes**	
Patients with available final echocardiography	28/31 (90.3)
Follow-up duration (months), median (IQR)	6.8 (1.9–18.2)
Reintervention (balloon angioplasty)	1 (3.2)
Reoperation	0

**Table 5:** Comparison Between Isolated Aortic Arch Surgery and Other Aortic Arch Pathologies.

Variable	Isolated Arch (*n* = 8)	Other Arch Pathologies (*n* = 23)	*p* Value
Cardiopulmonary bypass time (min)	97.0 (92.5–120.0)	130.0 (115.0–142.0)	0.105
Cross-clamp time (min)	42.0 (15.0–72.5)	52.0 (30.0–73.0)	0.774
Ventilation duration (h)	36.0 (33.0–36.0)	48.0 (24.0–114.0)	0.450
Hospital length of stay (days)	11.0 (8.0–12.2)	14.0 (10.2–17.5)	0.118

## Data Availability

The datasets generated and/or analyzed during the current study are not publicly available due to patient privacy and ethical restrictions but are available from the corresponding author on reasonable request.
